# Patients’ Experiences With Using a Digital Platform for Chat-Based Consultation in Primary Health Care in Sweden: Qualitative Study

**DOI:** 10.2196/77478

**Published:** 2025-08-13

**Authors:** Pär Eriksson, Felicia Gabrielsson-Järhult, Helén Thorold Nylin, Evalill Nilsson

**Affiliations:** 1 eHealth Institute, Department of Medicine and Optometry Faculty of Health and Life Science Linnaeus University Kalmar Sweden; 2 Department of Quality Improvement and Leadership School of Health and Welfare Jönköping University Jönköping Sweden; 3 Improvement and Innovation Unit Region Östergötland Linköping Sweden

**Keywords:** primary health care, telemedicine, health services accessibility, technology acceptance, patient satisfaction, thematic analysis

## Abstract

**Background:**

The growing demand for primary health care necessitates more efficient use of resources. Digital health solutions, such as online platforms for patient-provider communication, are seen as promising tools to enhance service delivery efficiency. However, adoption in Swedish primary health care remains limited. Understanding the factors that influence patients’ choices regarding digital health services is essential to realizing potential efficiency gains as these services scale.

**Objective:**

This study aims to explore patients’ experiences using the digital platform 1177-direkt for chat-based consultations in Swedish primary health care, with a focus on understanding their concerns when accessing services digitally.

**Methods:**

We conducted 23 semistructured interviews between March 2024 and December 2024 with patients from 3 health care regions in southeastern Sweden, focusing on their use of 1177-direkt to contact their primary health care center (PHCC). Thematic analysis was performed using an inductive approach. Additionally, a sentiment analysis was conducted, categorizing statements as positive, negative, or neutral. The unified theory of acceptance and use of technology (UTAUT) framework was applied to interpret the findings, given its widespread use in predicting technology acceptance in consumer contexts.

**Results:**

Three main themes emerged: (1) digital technology’s impact on access to services, (2) perceived digital platform functionality and usability, and (3) shifts in the patient-provider dynamic through digital communication. In addition, there were 9 subthemes. Patients expected quicker access to their PHCC via the platform, but this was often offset by delays later in the process. Most found the platform easy to navigate, though uncertainty remained about appropriate use cases. Concerns were raised about the automated symptom checker, which was seen as either too broad or too narrow and often failed to interpret or contextualize patient input—leaving users to draw their own conclusions. Patients, especially returning users, expressed a desire for more personalized interactions and perceived a tension between digital contact and relational continuity. Sentiment analysis revealed that infrequent users of health care responded more negatively than frequent users.

**Conclusions:**

This study highlights a wide range of user experiences, with most participants encountering both benefits and challenges. The sentiment analysis offered novel insights not commonly reported in similar research. To enhance the efficiency of digital platforms in primary care, key areas to address include ease of access, time-saving potential, and a more personalized user experience. Neglecting the needs of returning patients may hinder broader adoption of digital primary care services.

## Introduction

To meet the increasing demand for primary health care, providers must manage resources more effectively [1-4]. The adoption of telemedicine consultations and digital platforms for accessing services among providers has the potential to contribute to efficiency gains [5-7]. Scaling up and shifting to digital health care are key themes in core policy documents in Sweden and elsewhere [8,9]. What constitutes a reasonable share is debated, but 20% is often cited as a target [10]. However, uptake in primary health care in Sweden is lagging behind overall expectations. The number of patients who are using digital apps, websites, or patient portals to communicate with health care services is increasing [11], but the proportion of digital consultations between patients and health care professionals remains low [12]. Thus, predicting user behavior and understanding patients’ reasons for using or not using digital health services is critical. It could provide important information to policymakers about potential efficiency gains as digital health care is being scaled up.

Patient characteristics associated with higher use of digital health care have been described in several studies. These include younger age, being female, living in metropolitan areas, and high socioeconomic status [13-17]. In a Swedish context looking at users of digital care integrated into primary health care, geographical differences based on urbanicity were found not to affect utilization of digital care, but low socioeconomic status and high morbidity were found to have a negative effect on utilization [18]. In a similar study from two regions in the southeastern part of Sweden, users were reported to have low to moderate health care needs but also to generate more electronic health record (EHR) entries than nonusers [19]. Looking at patient-reported reasons for choosing digital health care, several studies have reported high accessibility and convenience as being essential [20-24]. Benefits for certain patient groups have also been described in several studies; for example, patients living with chronic conditions have been found to benefit from having an “open channel” with their provider [25]. Patients with mental health problems and neuropsychiatric disorders have been found to benefit from digital communication as they can have their consultation and remain at home in a familiar environment at the same time [26]. Similarly, patients experiencing depression and anxiety benefit from having the opportunity to explain their problems in writing and when their ailment is most severe [25]. This suggests that digital communication can be a useful tool when combined with continuity of care [27]. Results from Canada suggest that patients older than 45 years and patients with complex health problems were more likely to choose virtual visits with a known provider as opposed to a health care professional they had not met before and that patients with mental disorders were overrepresented in virtual visits compared with the general population [28]. Furthermore, results from a systematic review in 53 member states in World Health Organization (WHO) Europe indicated benefits with the use of telemedicine technology in the screening, diagnosis, management, treatment, and long-term follow-up of chronic diseases [29]. Similar results have been reported among users of telemedicine consultation in Sweden and patients with chronic obstructive pulmonary disease or asthma and depression [30]. Reasons for not using digital health services included strong preference for human-based health care [31], valuing seeing the same health care professional [24], low trust in digital services, not being able to afford the necessary equipment such as a smartphone or computer, and low digital literacy [31,32].

Understanding user behavior is essential for successfully scaling up telemedicine consultations in primary health care. To achieve this, the unified theory of acceptance and use of technology (UTAUT), developed by Venkatesh et al [33], can be a useful tool in the analysis of data. This model has been widely applied in similar studies to predict technology acceptance in consumer contexts [34,35]. UTAUT has identified 4 key constructs that influence user behavior: performance expectancy, effort expectancy, social influence, and facilitating conditions. It has proven effective in previous research on telemedicine and eHealth [33], making it a valuable and relevant tool for this study.

The Swedish health care system is divided into 21 autonomous regional health authorities, hereafter referred to as “regions.” Funding is tax-based. First-line care is provided through multiprofessional primary health care centers (PHCCs) offering a range of services to registered patients. Reimbursement in primary health care is based on a combination of capitation and fee-for-service. The way a person makes contact with their PHCC is normally by a queue-based phone service (TeleQ). Some PHCCs also offer drop-in services for registered patients. Other options for contact are available for anyone who wants to reach their PHCC via a web-based portal, which is called *1177.se*. This national portal offers e-services for renewing prescriptions, accessing EHRs, results of medical tests, nonemergency medical advice, information on health and diseases, and information about other health care services available in the Swedish health care system.

The newest addition to *1177.se* is a digital platform for consultation called *1177-direkt*, hereafter referred to as “the digital platform.” This platform offers patients the option of contacting their primary health care provider via a chat function using a smartphone, tablet, or computer. It offers an automated symptom checker followed by automated triage, directing the patient to an appropriate level of care and further action based on the patient’s reason for contact. This includes automated self-help advice or a chat with a health care professional, with the option to upload images and switch to a video meeting or phone call. Parents or guardians can use the service for their children until they have reached 13 years of age or for other relatives unable to use the services themselves. At the time of the study, the digital platform was operational in 11 of the 21 regions. Another 5 regions operated a similar service, making the digital platform the prevailing mode of digital contact in Swedish primary health care.

Understanding the factors that influence user behavior among patients is crucial for capturing potential efficiency gains in primary health care services. The aim of the study was therefore to explore patients’ experiences with using a digital platform for chat-based consultations to understand their concerns when contacting primary health care services online.

## Methods

### Study Design

We conducted a qualitative inductive interview study using thematic analysis combined with sentiment analysis to explore patients’ experiences with using the digital platform. Inclusion criteria were having used the digital platform at least once during 2024 and being available for an online interview in Swedish. Sentiment analysis was used to identify differences in user experience based on the frequency of using health care services [36,37].

### Setting

**Figure 1:**
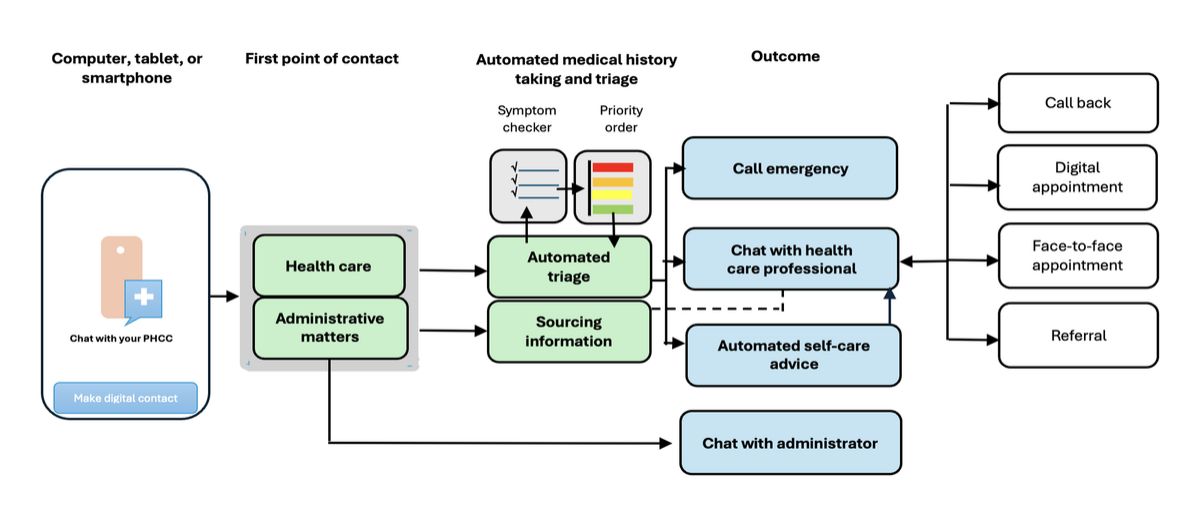
Case management in the digital platform adapted from local procedures (may differ between regions). PHCC: primary health care center.

Approximately 120 PHCCs serving 1.1 million residents in 3 regions in the southeast part of Sweden constituted the population from which participants were recruited. These 3 regions collaborated closely before and after the launch of the digital platform regarding technical optimization, training, and support. The digital platform was accessible via computer, tablet, or smartphone during the opening hours of the affiliated PHCCs, putting patients in touch with their own PHCC (Figure 1). In one of the regions, the digital platform was also staffed during evenings and weekends, but this was by a central regional unit. The platform was neutral in terms of registration, but patients could be referred back to their own PHCC, if necessary. Typically, a contact was initiated by the patient, either by choosing “Health care” or “Administrative matters” from the web portal. Following a patient-initiated contact, the patient was asked to complete an automated symptom checker, which comprised a set of predefined health concerns that generated predefined outcomes based on patient responses. In the subsequent automated triage, the information from the symptom checker was summarized and recorded in a table with a proposed order of priority. An assigned nurse staffing the platform assessed and managed the enquiry. Images could be uploaded by the patient, and additional information about the patient such as patient records could be obtained by the nurse. The nurse could make a second assessment and, if deemed necessary, readjust the order of priority before proceeding. Administrative matters were dealt with separately. In case a staff member was not available to deal with the enquiry immediately, a message would be sent to the patient informing them that the chat conversation would start later. The chat conversation could result in, for example, an appointment with a physician, if possible, the patient’s named accountable care contact (defined as the general practitioner officially assigned to a patient and responsible for that patient’s overall care within a PHCC) or other staff members at the PHCC relevant to the case. These appointments could either be online (chat conversation or video meeting) or at the PHCC. Staff members handled several enquiries on the digital platform simultaneously.

A total of 152,960 contacts have been made via the digital platform in the 3 regions since the launch in 2023 until the end of the study period in December 2024. This was an estimated share of 2% of the total number of contacts with PHCCs in these regions based on information in *Vården i siffror,* a national website providing current statistics from health care services all over Sweden [38]. Contacts include physical and digital visits. Administrative inquiries, laboratory tests results, or advice are not included. Of these contacts, 61% (92,999/152,690) were made by women, and 39% (59,961/152,960) were made by men (Table 1). The estimated mean age was 37 years. It was a phased introduction of the digital platform; all regions were fully operational by June 2023 [39].

**Table 1:** Contacts with primary health care in the 3 regions initiated through the digital platform (N=152,960) by region, sex, and age group from the launch until the end of the study period (January 2023-December 2024).

Characteristics			Region 1 (n=12,077)		Region 2 (n=76,587)		Region 3 (n=64,296)
**Sex, n**							
	Men (n=59,961)	4834		30,486		24,641	
	Women (n=92,999)	7243		46,101		39,655	
**Age (years), n**							
	0-9^a^ (n=14,818)	893		8676		5249	
	10-19^a^ (n=10,856)	833		5944		4079	
	20-29 (n=33,385)	2692		17,424		13,269	
	30-39 (n=31,337)	2514		15,321		13,502	
	40-49 (n=21,759)	1684		10,407		9668	
	50-59 (n=20,425)	1571		9258		9596	
	60-69 (n=12,313)	1109		5631		5573	
	70-79 (n=6377)	595		3055		2727	
	80-89 (n=1609)	180		821		608	
	≥90 (n=81)	6		50		25	

^a^Includes contacts made by parents or guardians.

### Participants

Patients who used the digital platform at least once, starting from January 2024, were asked if they would like to participate in the study. In 2 of the regions, triage nurses at 20 selected PHCCs were contacted and asked to invite patients to participate by having a nurse forward the invitation in the chat conversation at the end of the session. The selection of PHCCs to be approached was supposed to be purposefully sampled based on size, location, and socioeconomic index among the registered population. However, in the end, the selection of PHCCs was limited to those PHCCs that agreed to participate in the study. In the region where the digital platform had a central unit during out-of-office hours, the invitation was offered in the same way, regardless of the PHCC at which the patient was registered. During the recruitment of participants, strategic purposeful sampling was attempted. In the invitation from the nurse, the potential participants were asked to share their name and mobile telephone number, which were then forwarded to the research team who looked for characteristics such as sex and age in order to intentionally include heterogeneity in the group. The participants were then contacted by the research team via SMS text messaging and asked to provide an email address where further information about the research project could be sent. Two reminders were sent. All participants provided their oral informed consent, which was registered.

### Data Collection

A semistructured interview guide was developed for the interviews. The guide was compiled by the research team based on evidence from evaluations, reports, and scientific articles regarding similar digital platforms [21,22,25] and was structured around 3 sets of questions: experiences and expectations before using the digital platform, experiences when using the digital platform, and experiences and effects after using the digital platform (Multimedia Appendix 1). Participants were also asked to state age, sex, time of contact with the PHCC, and how often they had visited health care in the past 12 months; rate their digital literacy by choosing one of 6 response options regarding self-rated digital ability (excellent, good, moderate, fair, low, none); and to state whether they would recommend the digital platform to others or not. The numerical cutoff to be considered a frequent user was set at 5 or more contacts per year following the methodology developed by Smith et al [40]. Three of the authors (PE, HTN, FGJ) conducted the interviews. Even though the interviews were structured in line with the interview guide, the interviewers were actively seeking an open conversation in which the participants were free to raise issues not covered in the interview guide [41]. The interviews were conducted using standard video conferencing platforms (eg, FaceTime) via smartphone or computer, but only the audio files were saved and stored. All interviews were transcribed verbatim by a professional transcriber. After the analysis, the interview data were deidentified and given an interview person number based on the time and date of the interview.

### Analysis

Thematic analysis was used, as described by Braun and Clarke [41,42]. An inductive approach was used in which the themes and subthemes were derived directly from the data without any preconceived theories or expectations. Inductive thematic analysis is also referred to as bottom-up analysis as it starts with the data and builds up to themes. A thematic description of the entire data set was developed, maintaining a rich overall description of the predominant and important themes. This is a useful method for investigating under-researched areas or working with participants whose views on the topic are not known [41]. Three of the authors (PE, HTN, FGJ) read all the transcripts. After that, each identified sentences or phrases from their own interviews to form meaning units corresponding to the study aim. The first iteration of coding meaning units was carried out by PE. Every meaning unit was given a code that reflected the original content as much as possible. Codes were translated into English, while subthemes and themes were all formulated directly in English. Quotes were translated by PE. The translation of quotes came after the analysis was completed. Only those quotes that were selected for inclusion in the manuscript were translated, resulting in a total of 19 quotes. Translation was supported by Google Translate. The entire manuscript was revised by a professional language reviewer before submission. The results of the first iteration were jointly reviewed by 3 of the authors (PE, HTN, FGJ). The second iteration included a second reading of the interviews by PE. The codes were revisited, and subthemes and early main themes were suggested. A mind map was also generated by PE to support the process of grouping and refining the subthemes and themes.

The results of the analysis were discussed and commented on by PE, HTN, and FGJ during a first joint workshop. PE continued refining the analysis and presented revised results in a second joint workshop where preliminary major themes were formed and a more consolidated mind map was developed together with a revised report. Based on the outcome of the second workshop, PE continued consolidation and added sentiment analysis as a new feature. Each identified sentence or phrase was identified as being positive, negative, or neutral [43]. Following a lexicon-based method for sentiment analysis [44], positive experiences described satisfaction, benefits or, for example, recommendations to others to use the digital platform. Negative experiences described dissatisfaction, challenges, or unfavorable outcomes. Neutral experiences were neither explicitly positive nor explicitly negative. A ratio of positive to negative sentiments was created for each participant. A ratio was also created for the 2 groups of frequent and infrequent users of health care services to explore the differences between the 2 groups. A chi-square test was used to examine the differences in ratios. A box plot was used to visualize the distribution of differences in sentiment scores between frequent and infrequent users of health care services.

A further refined and developed report was discussed at a third and final workshop attended by the entire research team (PE, FGJ, HTN, EN). During the final workshop, the UTAUT was discussed and identified as a useful lens to better understand and contextualize the findings. The UTAUT constructs include performance expectancy, effort expectancy, social influence, and facilitating conditions. All constructs were used in the analysis. They were applied to support the interpretation of the themes, rather than guiding the initial coding process. Comments and suggestions were included in the final report. MS Excel for Mac 16.93.1 was used for analysis of qualitative data, and STATA 19.5 was used for quantitative data.

Braun and Clarke [41] emphasized the importance of trustworthiness during analysis, which refers to the credibility and reliability of the findings. Trustworthiness was strengthened by a continuous dialogue between the authors and their peers. The first author (PE) performed the analysis, assisted by HTN and supervised by FGJ and EN. An example of the analysis is provided in Table 2.

**Table 2:** Examples of meaning unit, sentiment, code, subtheme, and theme.

Interview participant (IP) number	Meaning unit	Sentiment	Code	Subtheme	Theme
IP6	I got in touch with the nurse quite quickly and received a phone call from the doctor later the same day. However, regarding the first contact during the chat, this happened quite quickly.	Positive	Quick response	Ease of access to care	Digital technology impacts on access to services
IP1	No, it is only the fact that it might be difficult to know what to choose if you have a designated care contact and an ongoing issue. Then it is not so obvious.	Negative	Designated contact at PHCC^a^	The conflict between digital contact and relational continuity	Shifts in the patient-provider dynamic through digital communication

^a^PHCC: primary health care center.

### Ethical Considerations

Ethics approval was obtained from the Swedish Ethical Review Authority (Dnr 2023-05555-01). Supplementary ethics approval was obtained to also include young people who are not of legal age but older than when their parents or guardians have full access to their health records (Dnr 2024-03480-02). Written information about the study was given to all the participants, and all of them gave their informed consent. No compensation was provided to participants for their participation. All data were kept on a secure server in accordance with Linnaeus University guidelines. The study was carried out in compliance with relevant data protection regulations, including the General Data Protection Regulation (GDPR).

## Results

### Participants

A total of 23 patients agreed to participate. Their ages ranged from 16 years to 70 years with a median age of 39 years, and 65% (15/23) were women (Table 3). Some were using the platform on behalf of their children. The 23 interviews lasted from 24 minutes to 48 minutes, and 648 quotes were identified and labelled as being positive, negative, or neutral. Three main themes, “Digital technology's impact on access to services,” “Perceived digital platform functionality and usability,” and “Shifts in the patient-provider dynamic through digital communication,” emerged, with 9 subthemes (Table 4).

**Table 3:** Characteristics of the participants.

Interview participant (IP) number	Sex	Age (years)	Time of contact with the PHCC^a,b^	Frequent^c^ or infrequent user of health care services^d^	Digital literacy^e^	Recommend digital platform to others
IP1	Woman	36	Office hours	Frequent	High	Yes
IP2	Woman	45	Office hours and out of office hours	Frequent	High	Yes
IP3	Man	70	Office hours	Frequent	Middle	Yes
IP4	Man	36	Office hours	Infrequent	High	Yes
IP5	Woman	36	Office hours	Frequent	High	Yes
IP6	Man	27	Office hours	Infrequent	Middle	Yes
IP7	Woman	23	Office hours	Frequent		Yes
IP8	Woman	48	Out of office hours	Frequent	High	Yes
IP9	Man	67	Out of office hours	Infrequent	High	Yes
IP10	Man	34	Out of office hours	Infrequent	High	Yes
IP11	Woman	41	Out of office hours	Infrequent	High	Yes
IP12	Woman	29	Office hours and out of office hours	Frequent	High	Yes, but not for people who are anxious
IP13	Woman	45	Out of office hours	Frequent	High	Yes, but not for older people
IP14	Woman	27	Office hours and out of office hours	Frequent	High	No
IP15	Woman	32	Out of office hours	Infrequent	High	No
IP16	Woman	39	Out of office hours	Infrequent	High	Yes
IP17	Man	49	Office hours	Frequent	High	Yes
IP18	Woman	16	Office hours and out of office hours	Frequent	High	Yes
IP19	Woman	17	Office hours and out of office hours	Frequent	High	Yes
IP20	Man	50	Office hours	Infrequent	High	Maybe
IP21	Man	40	Office hours	Infrequent	High	No
IP22	Woman	43	Office hours	Infrequent	High	Yes
IP23	Woman	55	Office hours	Infrequent	High	Maybe

^a^PHCC: primary health care center.

^b^Time of initiating contact with the PHCC via the digital platform (self-reported).

^c^≥5 times.

^d^Number of contacts with the PHCC during the past 12 months (self-reported).

^e^Self-reported ability: “High”=excellent and good, “Middle”=moderate and fair.

**Table 4:** Main themes, subthemes, description of subthemes, corresponding unified theory of acceptance and use of technology (UTAUT) constructs, and motivation or context.

Main theme and subtheme			Description of subtheme		Corresponding UTAUT constructs		Motivation or context
**1. Digital technology’s impact on access to services**							
	1.1 Ease of access to care	Participants’ experiences with contacting PHCC^a^ using the digital platform		Effort expectancy		Users expect the digital platform to make it easier for them as patients to access care	
	1.2 Establishing contact and communicating with the PHCC	Participants’ experiences with communication with the PHCC using the digital platform		Performance expectancy, social influence		Users expect the digital platform to enable easy and convenient communication with the PHCC, advice from others sometimes a factor	
	1.3 Availability of information about the digital platform	Participants’ experiences accessing information about the digital platform		Facilitating conditions		Availability of information about the digital platform constitutes a facilitating factor.	
**2. Perceived digital platform functionality and usability**							
	2.1 Perceived impact on user-friendliness	Participants’ concerns about the functionality of the digital platform and how it impacts efficiency and effectiveness for them as patients		Effort expectancy		Users expect the digital platform to be easy and convenient to use.	
	2.2 Automated symptom checker as facilitator of patients’ reasons for contact	Participants’ experiences with using the automated symptom checker		Performance expectancy, effort expectancy		User experiences reflect both the performance of the automated symptom checker and the effort required to use it.	
	2.3 User-friendliness of the interface	Participants’ experiences with navigating on the digital platform		Effort expectancy		User experiences reflect the effort it takes to understand and navigate on the digital platform.	
	2.4 Perceived impact on security in communication	Participants’ concerns regarding IT security		Facilitating conditions		IT security constitutes a facilitating factor.	
**3. Shifts in the patient-provider dynamic through digital communication**							
	3.1 Changing the terms of interaction	Participants’ experiences with interacting with health care professionals through the digital platform and how it affects the balance between health care providers and care recipients		Effort expectancy		User experiences reflect the change in effort depending on how the digital platform affects the balance between health care providers and care recipients.	
	3.2 The conflict between digital contact and relational continuity	Participants’ experiences with using the digital platform being returning patients		Performance expectancy		User experiences reflect expectations regarding performance of the digital platform among participants who are returning patients.	

^a^PHCC: primary health care center.

### Main Themes and Subthemes

Early in the process of forming the themes and subthemes, it became clear that the data were more scattered in content and meaning than expected. The respondents differed in opinions about similar experiences, and the same respondent might report both positive and negative sentiments regarding the same experience. The respondents commented on their own experiences but also on potential consequences for themselves as well as for others using the platform. The wide spectrum of opinions was not a problem but rather reflects the complexity of digital care. Opinions could differ depending on, for example, prior experiences, expectations, and health status. However, the spread created methodological challenges. Including the sentiment analysis resolved some of these challenges. The UTAUT model also provided a structure to better understand and appreciate the complexity of the data. During the analysis, 3 main themes and 9 subthemes emerged. Finally, each subtheme was linked to one or more of the UTAUT constructs.

#### Theme 1: Digital Technology's Impact on Access to Services

##### Overview

Main theme 1 describes the digital platform's impacts on access to services in terms of time, convenience, safety, and quality. Quick access to health care services and quick handling of enquiries were appreciated, but concerns related to integration of the digital platform with already existing services offered by the PHCC were raised by the participants.

##### Subtheme 1.1 Ease of Access to Care

The digital platform was perceived by several participants as creating improved accessibility. It contributed to easy and fast access to care regardless of time and space. Some participants made comparisons with how it was before the launch of the digital platform and perceived it as an improvement, for example, avoiding queues when contacting the PHCC via phone.

There’s such a long waiting time on the phone; this is much quicker...

IP11

Several participants appreciated the flexibility the digital platform offered, for example, communication via chat or video and image sharing. The design was also perceived to be helpful to quickly solve problems that the participants were facing; for example, administrative enquiries could be addressed through the digital platform in the form of chat.

On the other hand, the participants noted that, although the time it took to establish the first contact by logging into the digital platform, running through the automated symptom checker, and receiving a first message via chat was short, waiting time still occurred in the communication process that followed. Response times in the chat varied, and what constituted a “long time” was highly individual. Some participants questioned the added value of a digital contact when the waiting time exceeded their expectations and, in retrospect, felt that a phone call might have been more effective.

I initiated contact at 8 or 9 in the morning, and received a reply at maybe 10 that they would start the chat, and when the nurse had read my case and forwarded it to my doctor, he replied later that day.

IP1

References were also made by some participants to experiences with similar services offered by private direct-to-consumer providers, for example, providers specializing in digital care to families with small children. Switching between different providers was not uncommon.

We called [our health center], but there was such a long queue, so we turned to [a private provider specializing in digital consultation for parents with small children].

IP5

##### Subtheme 1.2 Establishing Contact and Communicating With the PHCC

Most participants expressed satisfaction with the fact that they could contact their PHCC via a digital platform and that digital contact with health care professionals was perceived as being in tune with the digital transformation in the rest of society. Nevertheless, some participants pointed to the importance of being able to communicate with a “real person” when contacting their PHCC and that it was sometimes difficult to distinguish between a “chatbot” and a “real person” when using the platform. Talking to someone on the phone or face to face about a concern created a sense of security that the enquiry would be dealt with. This type of security was more difficult to establish when using the digital platform.

...but then I still think it’s important that you can make a phone call as a patient and be able to talk to someone if you feel you have the need.

IP7

Some participants described their challenges and frustrations when making contact via the digital platform*.* For example, a situation was described where a concerned patient first contacted the PHCC by phone to request a physical examination at their PHCC. Once on the phone with the nurse, the patient was advised to hang up and contact their PHCC via the digital platform instead, in order to send the nurse a photo that could be assessed. Since the contact was made from a place where this was not easy to do, the patient would have preferred a physical examination at their PHCC.

I was probably a little frustrated at the time about having to take that detour and use [the digital platform] when I’d already made contact by phone. Why can’t I get help now?

IP23

##### Subtheme 1.3 Availability of Information About the Digital Platform

The participants were concerned about the fact that it was difficult to find the entry point to the digital platform. Some participants found it by chance, while others were referred to it or were invited by health care professionals, for example, when they made contact by phone. The entry point appeared to be hidden among several other *1177.se* services with similar names and was not clearly displayed. It was also not clear why this form of contact should be used compared with other types of contact. The participants expressed concern that, for example, older people would have difficulty using the digital platform because of this.

It wasn’t easy to find. I had to Google it to get directly to the page. I think it was very difficult to find it...

IP6

I was close to giving up and started looking for alternative options...

IP20

#### Theme 2: Perceived Digital Platform Functionality and Usability

##### Overview

Theme 2 describes the participants’ concerns regarding functionality and how it impacts efficiency and effectiveness for them as patients. The digital platform was perceived as saving time, being effective as it offered a user-friendly interface, and safe and secure. However, some participants perceived the automated symptom checker as cumbersome and difficult to use, which caused frustration among some. The fact that the chat often was a protracted process also caused frustration.

##### Subtheme 2.1 Perceived Impact on User-Friendliness

Efficiency in this context refers to the ability of the participants to maximize their time and resources when accessing health care services through the digital platform. Several participants perceived that having a chat conversation offered both convenience and discretion in their contact with their PHCC, for example, making it possible to combine the consultation with other household or workplace tasks, and that a chat conversation was also possible in a public place, at work, or in school, reducing the need to take time off work.

Precisely because of that flexibility that you don't need to step out and take a call...it can be sensitive information...that's the advantage of communicating in a chat.

IP14

Nevertheless, some participants expressed frustration because the chat conversation was not what they had expected. Instead of being a quick and efficient conversation, the chat could turn out to be a protracted process. This meant that, even if the initial response was quick, the participant could be left waiting for the next response over a long time frame and, in some cases, be disconnected due to inactivity. It also meant that the chat with the nurse could end up out of sync, leaving the participant feeling unsure about what was expected as the next step in the conversation. The participants were asking for better procedures in the chat conversation to make it more effective.

I felt that there was a bit of a delay in the response sometimes. That I had to wait and then I couldn’t leave the chat...so it became frustrating almost to the point that I thought I had lost the connection...Are they still writing?

IP14

On the other hand, the extended duration of the chat conversation was also perceived by some participants as allowing more time to reflect and prepare their responses. As a result, the chat felt less stressful than a phone call and contributed to a more positive experience when interacting with the PHCC.

##### Subtheme 2.2 Automated Symptom Checkers as Facilitators of Patients’ Reasons for Contact

As the automated symptom checker was the starting point for the process of establishing contact with the PHCC, it provoked many comments from the participants, the majority of which were negative. Many participants found it cumbersome and not always relevant to their problems. The symptom checker included a range of symptoms, which were often perceived as either too broad or too specific. The participants found it difficult to respond accurately and were concerned they would end up in the wrong line of questioning and not be able to retract. Several participants had to log out and start all over again, which caused frustration.

One participant also expressed concerns about the patient’s responsibility when answering questions in the symptom checker. It was perceived as difficult to assess the consequences.

...there were a lot of follow-up questions about do you have this or that and I felt at this point that I was in rather deep water as a patient. I cannot answer these questions. What kind of stuff am I answering? What will be the consequences?

IP21

Misunderstandings could also happen during the automated symptom checker. Some participants mentioned difficulties with conveying information about their condition and finding the right level of urgency to get the attention they wanted.

...in terms of volume, how much should I write to be taken seriously...?

IP6

The option to provide additional details through free text messages was not always helpful, as the word limit frustrated some of the participants. Nevertheless, participants appreciated the automated symptom checker, noting that it maintained a certain standard of quality in the process.

I think it’s quite convenient that you can enter what you’re looking for and then answer a set of questions that you might not have thought of yourself that might actually be important questions.

IP8

##### Subtheme 2.3 User-Friendliness of the Interface

Many of the participants accessing the digital platform via a computer or tablet found the interface to be user-friendly, although those participants who used the app on their smartphone were less satisfied. They also appreciated features such as image sharing with the PHCC and being able to retrieve the chat conversation after the visit and that these features were easy to use. Several participants believed that the user interface was more rewarding for younger patients and for patients with simpler ailments.

I don't think it was that difficult, it was very simple and clear how to do it.

IP19

However, a few participants who identified themselves as experienced users of digital technology and conversant in the digital world found the interface very hard to understand and not useful for them in their situation. They found the information confusing and not easy to follow. All these participants were infrequent users of health services.

I clicked on “Advice” but then, as I was unsure, I e-mailed the doctor instead to find out whether it was correct. Because I didn't know what “Advice” meant here.

IP20

##### Subtheme 2.4 Perceived Impact on Security in Communication

Most participants were satisfied with the IT security surrounding their contact via the digital platform. For example, they referred to the requirement for secure electronic identification. They trusted that all information would be stored securely and that consultations via the digital platform would not pose additional risks. Nevertheless, the participants also acknowledged the more general risk that all IT systems can be hacked and that digital information can be stolen. The issues of misinterpretation of their EHRs and the risk that it could adversely affect them in future communication with the health care services were also raised.

What kind of information do I give and how is it treated? If I press the wrong button here now, it will appear in my medical records that I have indicated something here that I have no problem with...

IP21

#### Theme 3: Shifts in the Patient-Provider Dynamic Through Digital Communication

##### Overview

The main theme 3 explores how digital communication reshapes the balance of interaction between patients and health care professionals. The participants observed a shift in responsibility from health care professionals to patients. Although some participants found it easier to express their concerns through the digital platform, others found it more challenging. Tension between digital contact and continuity of care was perceived as problematic.

##### Subtheme 3.1 Changing the Terms of Interaction

Contact via chat was appreciated by several participants who mentioned that a chat conversation involves a shift in power dynamics between patients and health care professionals in favor of patients. Being able to formulate both questions and answers in writing was mentioned as something positive that contributed to reduced stress but also gave the patients more confidence. This was especially appreciated by those participants who often felt that their symptoms were not taken seriously by health care professionals or participants who felt embarrassed about their condition. One participant described it as if they were “hiding behind the keyboard” and that they felt encouraged by that. The chat conversation neutralized and evened out a perceived power imbalance between patients and health care professionals.

The chat benefits those who don’t feel comfortable talking on the phone and gives me time to think before answering.

IP12

However, some participants also experienced a shift in responsibility from health care professionals to patients in interpreting symptoms, which can be challenging for some patients, contributing to a sense of the patient taking on more responsibility than what is usually the case when it comes to explaining their condition.

...it puts a lot of personal responsibility on the patient to be able to interpret their symptoms correctly and not everyone can do it right...People might be more worried than they should be - unnecessarily, I think.

IP12

##### Subtheme 3.2 The Conflict Between Digital Contact and Relational Continuity

Some participants, particularly returning patients, expressed concerns about how the digital platform aligned with expectation for relational continuity. A key issue was that the digital platform did not align well with having a named accountable care contact. This made it difficult for returning patients. The participants perceived that a contact through the digital platform was treated as a new, standalone interaction, regardless of their previous engagement with their PHCC. As a result, even returning patients would be treated as a first-time visitor, having to explain their entire health situation again, even though they knew this information was already known by the PHCC.

Not optimal if you have an ongoing case, where a new symptom assessment is not needed. Can't choose “contact with your designated care contact.”

IP1

Some of the participants who were returning patients described workaround solutions. For example, a “case” could be kept open on the digital platform, allowing patients to bypass the initial steps when making contact although, according to protocol, their case should have been closed.

For me, it’s very important because if it was the case...I would have to write about all the symptoms again. I don't do that now that we have my case open all the time...

IP13

### Sentiment Analysis

**Figure 2:**
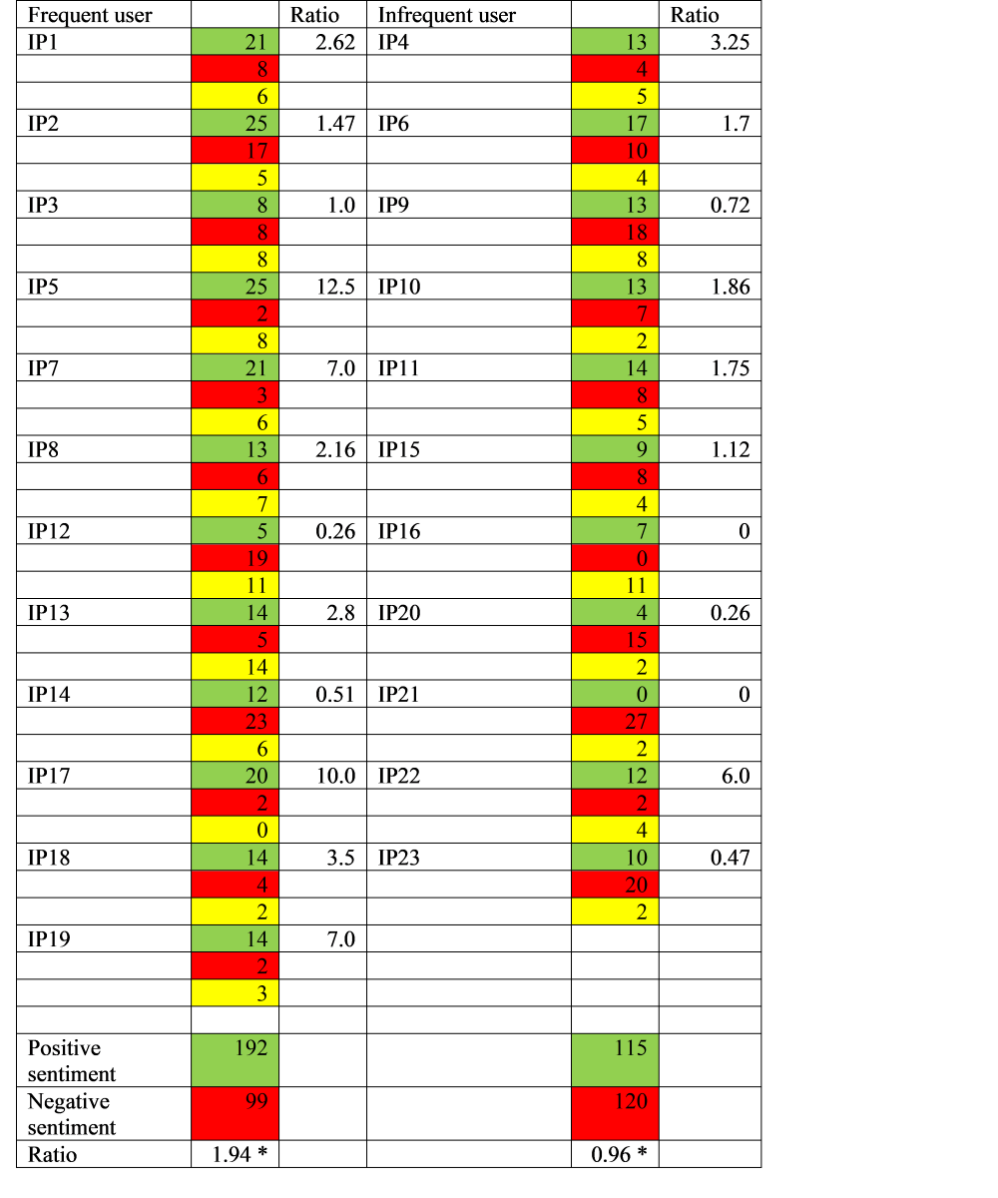
Color-coded sentiment score and ratio between positive and negative sentiments expressed in meaning units, by the frequent and infrequent use of primary health care services. Green: positive sentiment; red: negative sentiment; yellow: neutral sentiment. *Statistically significant at *P*<.05.

**Figure 3:**
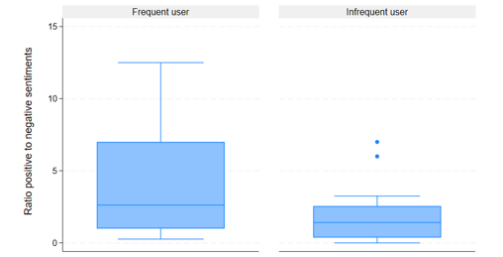
Box plot of differences in sentiment score between frequent and infrequent users of health care services.

All of the 648 quotes were coded for sentiment: positive, negative, or neutral. Of the total number of identified meaning units, 307 were categorized as positive, 219 were categorized as negative, and 122 were categorized as neutral. A sentiment score was then calculated for each participant (Figure 2). To explore differences between the 2 groups of frequent and infrequent users, a ratio of positive to negative sentiments was calculated for the 2 groups (Figure 2). The ratio of positive to negative sentiments among frequent users was 1.94, while the ratio of positive to negative sentiments among infrequent users was 0.96. This means that frequent users were more positive in their comments related to their experiences using the digital platform than infrequent users. This difference was found to be statistically significant (n=526; χ²_1_=19.08, *P*<.001). Furthermore, a box plot was used to compare the distribution of the data (Figure 3). It indicates a wider spread among frequent users than among infrequent users, meaning that participants in this group expressed both positive and negative sentiments. Among the group of infrequent users, the spread is narrower, and participants expressed mainly negative sentiments.

## Discussion

### Principal Findings

This study is one of the few studies to investigate patients’ experiences using a digital platform for chat-based consultations between patients and health care professionals in primary health care services in Sweden.

Overall, the participants appreciated the quick access and swift handling of enquiries through the platform. Most of the participants found the interface to be user-friendly, especially when using a laptop or computer as opposed to a smartphone. Contacting the PHCC using the platform offered discretion and convenience, which was perceived as added value for the participants. The digital contact was also perceived as changing the balance of interaction between patients and health care professionals in favor of the patient, making it easier for certain patient groups, such as patients who felt embarrassed or experienced prejudice about their condition, to get in touch with their PHCC. In this study, IT security was not found to be a major issue. Easy access to EHR notes from the conversations was appreciated.

On the other hand, the participants found it challenging to know when the platform would be their best option, compared with other means of contact. The automated symptom checker was perceived by many participants to be cumbersome and not suitable for its purpose. For some participants, the range of symptoms was too broad, while for other participants, it was too specific and therefore did not offer the support they expected. Participants perceived a conflict between contact through the digital platform and relational continuity. It was perceived as negative and counterproductive to continuity of care. The sentiment analysis of the responses showed that infrequent users of health services were, overall, more negative than frequent users.

### Limitations

Due to the small number of PHCCs participating in the study, the risk of selection bias, which affects transferability and representativeness of the findings, is acknowledged. Ultimately, however, the participating PHCCs exhibited a satisfactory variation in size, location, and socioeconomic status in the catchment area, which contributes to greater reliability in the results. Another limitation is that the study primarily included patients who were able and willing to use a digital platform when contacting primary health care. Patients who are less knowledgeable about digital technology or who have functional impairments were not represented. Their experiences and perspectives are important and may differ from those captured in this study. However, including under-resourced groups would require a different type of recruitment process. Studies investigating under-represented and under-resourced groups (eg, low socioeconomic status, people with impairments, older adults) and patients’ reasons for not using a digital platform for contact if such a platform is available should therefore be encouraged. Such studies can complement our findings and could further deepen the understanding of acceptance and adoption of digital technology such as a digital platform for contact in primary health care.

### Comparison With Prior Work

Much of what we know about digital health care today emphasizes the importance of a person-centered approach that tailors services to the patient’s specific need, rather than relying on standardized solutions to address simple problems [20,22,24,45,46]. In a systematic review, Ladd et al [47] concluded that system issues, particularly relating to triage and access, affected the patient’s ability to sustain relational continuity when using digital platforms for contact with their health care provider. This could cause frustration among patients and ultimately lead to inefficiencies in the provision of care. These results are consistent with the findings in this study. In a study from Canada looking at the impact of virtual visits on primary care use, McGrail et al [28] found that virtual visits are used for less complex issues and therefore appear to complement rather than replace in-person visits. McGrail et al [28] also concluded that, although the digital format improves accessibility, particularly for patients who prioritize convenience, it may be less suited to those requiring patient-centered care. A careful integration into existing systems and structures with a focus on continuity of care is therefore required.

To better understand the factors influencing patients’ use of digital health care services in this study, the discussion was structured using the UTAUT model [33]. This model is useful for examining how performance expectancy, effort expectancy, social influences, and facilitating conditions shape user behavior and the adoption of digital technology in health care [48].

### Performance Expectancy

Performance expectancy is the degree to which using technology will provide benefits to users when they perform activities. Regarding performance expectancy, this study indicates that participants value quick and easy access [22,23,49]. The participants expect that contact via the platform will provide a quicker and more convenient path to their PHCC [20,21,23]. For some participants, it did, but the study also indicates that the perceived quick access was challenged when the actual waiting time was not necessarily shorter, rather just transferred downstream. Therefore, participants perceived the actual time it took from initiating contact and completing the consultation to be longer than what they had expected. This is an important finding, as it may negatively influence patients’ willingness to use similar digital platforms. For some, it introduces a sense of uncertainty, leading to skepticism about the effectiveness of the services.

Furthermore, the results highlight patient expectations for more personalized contact, particularly for returning users. The standardized protocol for accessing services through the digital platform did not facilitate such personalization. Although it remains unclear whether this discouraged patients from using the platform, there is an obvious risk that potential efficiency gains could be lost if this is not addressed. Given the platform’s low uptake, this warrants careful consideration.

As aforementioned, most participants expressed both positive and negative sentiments regarding the same experience. Therefore, a sentiment analysis was conducted, to look for patterns in data that could shed more light on the experience. The results indicate that frequent users of health care services were more positive in their responses than infrequent users. These findings are interesting and have not been reported in other similar studies. Patient satisfaction has been found to be closely linked to initial expectations and subsequent perception of performance [50]. It is reasonable to assume that returning patients have expectations that are adjusted based on their previous experiences. Therefore, preferences of returning patients should be carefully considered to achieve efficiency gains from adopting a new technology. On the other hand, it has been reported that patients who value continuity of care are more likely to seek physical care rather than use a digital platform [24]. Similarly, older patients who wanted their requests to be handled by a known health care professional have been found to be less prone to use a digital platform [23]. Failure to recognize the needs of the group of returning patients could reduce the level of uptake [48].

### Effort Expectancy

Effort expectancy describes the degree of ease associated with the use of the digital platform. Most participants found the digital platform to be user-friendly and easy to navigate. The participants also expected the contact via the digital platform to be convenient in that it would enable a consultation from home or at work and that it would give them more autonomy compared with other forms of contact. Time to reflect before replying in a chat conversation was also mentioned as something positive.

Effort expectancy was also affected by the fact that the participants found it difficult to understand the kind of situations and enquiries for which the digital platform should be used. Combined with some participants finding it difficult to access the digital platform, this could negatively impact their willingness to use it.

Another concern was the automated symptom checker and the extent to which it was perceived as relevant to the enquiry placed by the patient [22,25,47]. The fact that the automated symptom checker had to be completed before a chat conversation could start caused frustration and sometimes discontinuation among participants. It must therefore be assumed that effort expectancy was negatively affected. The automated medical history-taking was not perceived as supportive when the patient made contact via the digital platform but was rather perceived as an obstacle. It was also perceived as blunt in the sense that it did not interpret and contextualize patient responses but expected the patient to make their own interpretation. Perhaps this is what comes across in the results, when patients stated that they felt uncomfortable not knowing what their responses would lead to next and that they felt that the responsibility was with themselves as patients to interpret their problems. On the other hand, an automated symptom checker can also act as a form of quality control, ensuring that standard questions are asked, which has been found in previous studies [25,27]. This was also acknowledged by some of the participants in this study, and for them, it might improve effort expectancy.

### Social Influence

The UTAUT model also includes social influence as an important factor for behavioral intention. This is about users adopting the digital platform due to personal choice, recommendations, or perhaps social pressure. Some participants felt obliged to use the digital platform based on recommendations from their health care provider, even though they themselves did not see any obvious benefits. This is an interesting finding that has not been as prominent in earlier studies. It reveals a complex dynamic between health care providers’ recommendations and patient perceptions, suggesting that, although endorsement from providers can drive adoption, addressing patient concerns and ensuring clear communication about the benefits are crucial for ensuring sustained engagement.

### Facilitating Conditions

Facilitating conditions are about perceptions of the resources and support available to perform a behavior. In this study, using a computer or tablet was found to be easier for the participants than using a smartphone, and access to and familiarity with how to use these devices were found to be facilitating factors. Most participants scored high on self-reported digital literacy. This is not surprising given that Sweden scores high on digital literacy in general. In 2023, the Swedish Internet Foundation reported that 94% of the population was using the internet on a daily basis [51]. On the other hand, some participants experienced a lack of technical support, which, in some cases, seems to have negatively affected their behavior. For example, some participants discontinued contact and switched to other forms of contact (telephone or physical visit) or even turned to other digital care providers. Other studies have found that digital communication between patients and health care professionals is subject to certain limitations, such as the patients’ level of knowledge [24,31] as well as health care professionals’ competence and ability to master the “digital encounter” [20,27]. Different types of user support integrated into the digital platform, clarifying when and how to best use it, could potentially solve some of the problems identified.

### Behavioral Intention and User Behavior

As the uptake of digital health services among users of primary health care is low, innovations such as digital platforms will only have limited effects on efficiency. During the study period, the proportion of contacts via the digital platform in the 3 regions was only about 2% of the total number of contacts. A key issue for health policymakers should be to identify where potential efficiency gains can be found and how digital health technology can capture these gains. This study suggests that frequent users have a more positive attitude toward the digital platform than infrequent users. It is reasonable to assume that these frequent users often include returning patients and those who require continuity of care. A digital platform design that does not prioritize the needs of this group risks missing important opportunities for improving care as well as for achieving much-needed efficiency gains in primary health care.

### Conclusion

This study reveals a wide variation in user experiences, with most participants encountering both opportunities and challenges. A sentiment analysis provides more insights into user behavior not reported in similar research earlier. For digital platforms to improve efficiency in primary care, the following key areas should be considered: ease of access, time-saving aspects, and a more individualized contact process. Failure to recognize the needs of returning patients may hinder higher uptake of digital primary care.

## Appendix

Multimedia Appendix 1 Interview guide 1177-direkt.

## Abbreviations

EHR: electronic health record
GDPR: General Data Protection Regulation
PHCC: primary health care center
UTAUT: unified theory of acceptance and use of technology
WHO: World Health Organization

## Data Availability

The data set analyzed during this study are not currently available due to privacy or ethical restrictions, in line with the approval of the Swedish Ethical Review Authority.
