# Supplementary material for: Patients’ Experiences With Using a Digital Platform for Chat-Based Consultation in Primary Health Care in Sweden: Qualitative Study
Source: J Med Internet Res. 2025 Aug 13;27:e77478. doi: 10.2196/77478 (PMC12401068; doi:10.2196/77478)
Supplement: Multimedia Appendix 1 [file jmir_v27i1e77478_app1.docx]

**INTERVIEW GUIDE – 1177-DIREKT**

**Purpose:** To capture perceptions of **accessibility, security, and individual customization**

**Welcome to this interview: Name**

**Give the participant information and ask for consent to participate**

**Interview Questions**

**1. Experiences, Expectations, and Concerns Before Use**

- This interview is about the new digital healthcare service, **1177 Direkt**. How did you find out that the healthcare service in your region launched 1177 Direkt?
- Are you registered with a health center? Which one?
- When you previously wanted to contact the healthcare system/your health center, how did you do it? Can you describe your previous experiences—how did those contacts work for you?
- Have you needed healthcare in the past year?

**Follow-up questions:**

- What type of care? Contact with a health center/primary care, specialist/hospital care, or a digital provider?
- Approximately how many healthcare contacts have you had over the past year?
- When you contacted healthcare through 1177 Direkt, was it on your own initiative or were you recommended to do so by someone? Who, or how?
- Do you remember your thoughts or reasoning when you chose this way to contact healthcare—i.e., through 1177 Direkt? (pros and cons)
- When you have sought healthcare previously, have you used other digital services/apps? For example, 1177 medical advice line by phone.
- Are you aware that you can read your medical records online via 1177? Do you usually use or have you used that service?
- Have you used other chat services with healthcare providers—private or public, e.g., 1177, or services like “xxx”?
- If you were to estimate your familiarity with using digital services/IT tools, where on the scale would you say you fall—from beginner to very experienced?
- Do you need help from someone else when using them?
- **Possible follow-up:** Would you like to share who helps you—child/grandchild/friend/other?

**2. Experiences During Use**

**User-friendliness – comprehensibility**

- Can you describe how the visit/contact went?
- How did you perceive the design—was it easy for you to find and understand how the service works?

**Accessibility**

- Do you remember roughly what time of day (office hours/after-hours) and what day of the week you made contact/started your case? If you’ve done this multiple times, did you notice any differences?
- How long did it take before you received feedback/a response? Did you get a reply in real-time or did the case remain ongoing for a while? Can you estimate how long it took in total for your case to be resolved?
- Have you used the service on behalf of someone else, e.g., an elderly relative/child?
- Did you also seek care in another way for this case? When and how?

**Efficiency – value creation**

- Did you feel that you received the help you needed?
- Have you used the service several times/for different matters? Have these contacts worked well or not so well for different types of cases? For example, a more acute case needing advice/assessment, or an administrative issue like a prescription?
- Have you previously used 1177’s medical advice line by phone, speaking with a nurse? And/or received advice from your health center by phone?
- If yes, how does that experience—speaking to a healthcare professional—compare to writing/chatting?

**Security**

- Did you feel confident that the information you entered was handled securely—technically speaking (data security)? BankID or other e-identification?
- What are your thoughts on the safety of the medical assessment you received (i.e., patient safety)?
- Did you have the opportunity to describe everything you needed, so that staff had the information they needed to make a correct medical assessment?

**3. Experiences of Outcomes and Effects After Use**

- Do you feel that 1177 Direkt affects your need for additional healthcare contacts? If yes, how?
- Based on your experience, who do you think this service is suitable for, and for what type of cases?
- Have you spoken to anyone else who has used 1177 Direkt, or otherwise heard about others' experiences using this service?
- Does this service save time and money—or the opposite?
- What was best—what advantages made the service meet your needs?
- Do you see anything that could be improved?
- Does the service add value for you? What are your thoughts on this service as a complement to other healthcare services you use? Pros/cons?
- Cost: Did you receive information about the cost of using 1177 Direkt as a patient? When/how did you receive this information?
- Would you recommend someone else to use the service? If yes, who?
- Is there anything else you’d like to add?

**Ask if the interviewee wants to receive the results/report.**

Thank you!
